# A Comparative Study on the Mechanical Properties and Microstructure of Cement-Based Materials by Direct Electric Curing and Steam Curing

**DOI:** 10.3390/ma14237407

**Published:** 2021-12-02

**Authors:** Zhihan Yang, Youjun Xie, Jionghuang He, Fan Wang, Xiaohui Zeng, Kunlin Ma, Guangcheng Long

**Affiliations:** School of Civil Engineering, Central South University, Changsha 410075, China; jiandan0115@gmail.com (Z.Y.); xieyj@csu.edu.cn (Y.X.); hjh2017@foxmail.com (J.H.); wn2020@csu.edu.cn (F.W.); zxhzlh@126.com (X.Z.); Makunlin@csu.edu.cn (K.M.)

**Keywords:** direct electric curing, steam curing, mechanical properties, microstructure, Joule heat, energy consumption

## Abstract

Direct electric curing (EC) is a new green curing method for cement-based materials that improves the early mechanical properties via the uniform high temperature produced by Joule heating. To understand the effects of EC and steam curing (SC) on the mechanical properties and microstructure of cement-based materials, the mortar was cured at different temperature-controlled curing regimes (40 °C, 60 °C, and 80 °C). Meanwhile, the mechanical properties, hydrates and pore structures of the specimens were investigated. The energy consumption of the curing methods was compared. The results showed that the EC specimens had higher and more stable growth of mechanical strength. The hydration degree and products of EC samples were similar to that of SC samples. However, the pore structure of EC specimens was finer than that of SC specimens at different curing ages. Moreover, the energy consumption of EC was much lower than that of SC. This study provides an important technical support for the EC in the production of energy-saving and high early-strength concrete precast components.

## 1. Introduction

Steam curing (SC) is a commonly used curing technology for concrete precast element production. SC uses water vapor temperature to raise the environmental temperature, then accelerates the hydration process of cement-based materials [1,2], which promotes the growth of mechanical properties [3,4] and forms the morphology and characteristics of microstructure [5,6,7,8]. SC shortens demolding times and increases productivity, which improves economic efficiency. However, many studies have suggested that SC has some adverse effects on specimens, such as the coarsening of pores [9,10], thermal damage leading to cracking [11,12], reduction of long-term performance [13], and reduction of durability performance [14]. In response to the advantages and disadvantages of SC, a new direct electrical curing (EC) [15] method has been developed by research scholars. EC uses fresh specimens with low resistivity [16] to generate uniform Joule heat under the current to accelerate the hydration of cement and enhance early mechanical properties. Existing studies mentioned in the literature [17] include the feasibility of EC application [18,19], field application [20], electrodes arrangement [21], curing regime [22,23], effects of hydration process and microstructure [24,25], mineral admixtures [26], durability [16], etc. The current mainstream research direction mainly uses Joule heating to solve the problem of winter construction at ultra-low temperatures [27,28,29,30], repair and reinforcement of building structures [31], and strengthening concrete [32,33,34]. These studies have proven that EC has the advantages of shortening the curing time, improving the early mechanical properties of cement, wide application prospects, and low energy consumption. The disadvantages of EC include thermal damage to the specimens caused by high temperature, pore coarsening, and long-term performance degradation.

As a newly rapid curing method, there is a lack of information related to the influence of EC on the properties and structures cementitious materials. In particular, there is no comparative study on the effect of cement mortar. To understand the effects of SC and EC on cement-based materials under the same curing regime, this paper investigates the effects of SC and EC on the mechanical properties and microstructure of cement mortar, with special attention to the corresponding mechanisms and energy consumption. This research aims at providing a technical support for the production of low-energy and early high-strength precast components applying the EC method.

## 2. Materials and Experiments

### 2.1. Materials and Equipment

P⋅I 42.5 (according to GB/T 175-2007) cement was used in this study, which was made by grinding cement clinker and gypsum without any supplementary cementitious materials. The chemical of cement was determined by XRF, and the mineral composition of the clinker were analyzed by the Bogue method. The details are shown in Table 1. The sand used was river sand with a grain size of 2.8 mm. A cement mortar mold with a size of 40 mm × 40 mm × 160 mm was used in this experiment.

**Figure 1:**
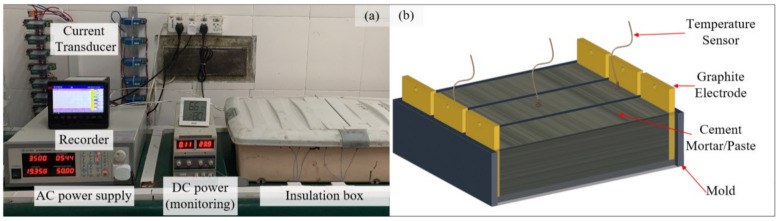
Diagrams for EC equipment (**a**) and specimens (**b**).

The equipment used and the arrangement are shown in Figure 1a. The laboratory-made insulation box and alternative current (AC) power supply was used to cure the EC specimens. The insulation box was composed of an external sealed plastic box and an internal aluminum foil sponge patch. The AC power supply model (APS-4000C, IVYTECH, Hangzhou, China) had a voltage range from 0 V to 300 V and a frequency range from 45 Hz to 250 Hz. High-precision anti-interference temperature sensors (WZP-PT100, Sinomeasure, Hangzhou, China) and AC transducers (SIN-SDJI-B-500mA, Sinomeasure, Hangzhou, China) were employed to detect the temperature and current of the specimens. The tested data were input to the multichannel paperless recorder (SIN-R6000F, Sinomeasure, Hangzhou, China) for recording. In addition, graphite electrodes and silver-plated wires were used in this study to guarantee high electrical conductivity and stability. They did not react with the cement paste, which avoided the change of hydrate phase and reduced the losses of energy during the current transmission. The schematic diagram of the specimen during the curing regime is shown in Figure 1b. The water bath tank provided a curing space with 600 mm × 300 mm × 200 mm for the SC specimens and its heating power was 2000 w.

### 2.2. Experimental Methods

In this study, mortar was prepared to test the strength and macropore characteristics. Meanwhile, pastes were conducted to explore the hydration degree, hydration products, and microstructure.

#### 2.2.1. Casting and Curing

In casting and curing, two graphite electrodes with a size of 40 mm × 50 mm × 5 mm were placed on both ends of the molds and fixed with hot melt adhesive. The mass ratio of cement to sand for the mortar sample was 1:2, and the water-cement ratio was 0.35. The cement and sand were placed in a mortar mixing pot and stirred for 1 min to ensure even mixing. After adding the water, the mixing regime included slow mixing (150 r/min) for 2 min and fast mixing (300 r/min) for 2 min. After mixing, the cement mortar was poured into the molds, and then a vibrating table was used for compacting the mortar with two 5 s compaction sessions. A polyethylene film was used to seal the surface of the specimens to prevent water evaporation after vibrating. Before the start of the curing regime, the EC and SC specimens were placed into the homemade insulation box and water bath tank, respectively, and the temperature sensor was inserted into the positive center of the specimen.

**Figure 2:**
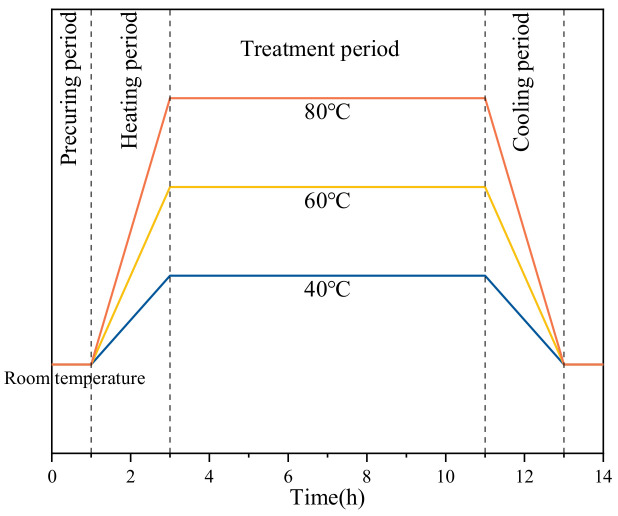
Schematic diagram of the curing regimes on this experiment.

The curing regime was divided into four stages as shown in Figure 2, including the precuring period (0–1 h), heating period (1–3 h), thermostatic period (3–11 h), and cooling period (11–13 h). The rate of temperature rise in the three curing regimes was 10 °C/h, 20 °C/h, and 30 °C/h, and the temperature in the constant temperature stage was 40 °C, 60 °C, and 80 °C, respectively. Voltage was applied to the EC specimen in the heating period. When entering the constant temperature period, only 0.5 V was applied to the cement paste to reduce the Joule heat due to the hydration heat release. As the hydration heat was reduced, the applied voltage must be raised to increase the Joule heat to maintain the temperature of the specimens. At the end of 11 h of curing, the specimens were cooled down in a controlled manner after the AC power was turned off. The same curing regime was also used in the SC, and the difference was that the SC specimens were controlled by the temperature of water vapor in the water bath tank. In this paper, three curing regimes (40 °C, 60 °C, 80 °C) were selected, and three groups of specimens with different curing methods were formed. The specific specimen parameters are shown in Table 2.

#### 2.2.2. Testing

The specimens were demolded immediately after the curing regime and then placed into the standard curing room (20 ± 2 °C; 95 ± 5% RH). The specimens were taken out from the standard curing room at the corresponding curing age and left to dry naturally for 2 h before the compressive and flexural strength test. The broken samples were left for microscopic testing. Three specimens were tested the flexural strength, and then six broken specimens were used to test compressive strength test. The loading rate was kept at 50 N/s during the flexural strength tests and 2.4 kN/s during the compressive strength test. The average strength of the three specimens was used as the representative strength value. After the mechanical tests, the suitable samples were selected for the hydration process and microstructure tests, including thermogravimetric analysis (TGA), X-ray diffraction analysis (XRD), scanning electron microscopy (SEM), mercury intrusion Porosimetry (MIP), and macro/mesoscopic pore image analysis (MIA) measurements.

The chemical bound water and portlandite content were tested by TGA. The samples were vacuum dried after 7 days of isopropyl alcohol exchange to terminate the hydration and dry the samples. Then, the samples (approximately 50 mg powder) were tested from room temperature (RT) to 950 °C at a heating rate of 10 °C/min under a N_2_ atmosphere by equipment of TGA 2(SF)-Mettler Toledo. The loss in mass of samples from RT to 550 °C was determined as the chemical-bound water. The weight loss corresponding to the decomposition peak (about 400–550 °C) of portlandite was used to calculate the content of portlandite. The specific calculation formula for $\mathrm{Ca}{\left(\mathrm{OH}\right)}_{2}\;$content of samples is as follows:

(1) $$\mathrm{Ca}{\left(\mathrm{OH}\right)}_{2,measured}=WL_{\mathrm{Ca}{\left(\mathrm{OH}\right)}_{2}}\times \frac{M_{\mathrm{Ca}{\left(\mathrm{OH}\right)}_{2}}}{M_{H_{2}O}}$$

where $\mathrm{Ca}{\left(\mathrm{OH}\right)}_{2,measured}$ is the $\mathrm{CA}{\left(\mathrm{OH}\right)}_{2}$ content of samples; $WL_{{\mathrm{Ca}\left(\mathrm{OH}\right)}_{2}}$ is the weight loss from decomposition of portlandite; and $M_{{\mathrm{Ca}\left(\mathrm{OH}\right)}_{2}}$ and $M_{H_{2}O}$ denote the molar masses of $\mathrm{CA}{\left(\mathrm{OH}\right)}_{2}$ and $H_{2}O$, which are 74 g/mol and 18 g/mol, respectively.

The solid phase was analyzed by XRD. An X-ray diffractometer (X’Pert pro, Sibaiji, Shanghai, China) produced by Malvern Panalytical was used to collect the X-ray diffraction patterns. The diffractometer equipped a CuKα source at 45 kV and 40 mA. Soller slits of 0.04 rad fixed at 0.5°, a beam knife, and an X’Celerator linear position-sensitive X-ray detector with a length of 2.122° (2θ) were used for the X-ray diffraction measurements. Samples were scanned from 5° to 65° (2θ) at the rate of 0.02° (2θ) per step, at a scanning speed of 0.5 s per step, for a scan duration of about 30 min.

The pore structure of cement paste samples was tested by MIP and MIA. MIP samples of 20mm in length and 12mm in diameter were prepared and soaked in isopropanol for 7 days. They were then transferred to a vacuum-drying oven for another 7 days and tested using Autopore IV-9500 (Micrometrics, Shanghai, China).

The MIP machine was set to equilibrate for 10 s at each pressure point. The contact angle was 130°, and surface tension was 0.485 N/m. The MIA measurement was conducted by taking photos of different locations on the surface of the specimen using microscope magnification, identifying the air holes in the photos by software, and calculating the size and area of the air holes in the specimen using C++ programming language. Three cement mortar specimens were split in the middle of the length direction by a cutting machine. The cut pieces were selected as the observation surface and ground smooth by the grinder three times. The specimens were ground for 800, 100, and 1200 meshes using silicon carbide powders. Then, the black marker was used to black out the observation surface of the specimens. After the surface was dry, the heated zinc oxide-Vaseline pastes were poured on the surface to let it flow into the pores in the observation surface. After cooling to the room temperature, the excess pastes were scratched off with a scraper. Six specimens were placed under the microscope camera for clear observation. The white pixels in the image were automatically recognized as aperture by the computer.

In total, 225 microscopic images were captured for each observation surface by scanning along X and Y axis 15 times, respectively, starting from the upper left corner of sample. The porosity and pore diameter distribution were then automatically calculated through an algorithm realized by C++ programming language according to the following equations:

(2) $$A_{i}=\sum_{j=1}^{m_{i}}A_{j}$$

(3) $$D_{j}=\sqrt{\frac{4A_{j}}{\pi }}$$

(4) $$A\%=\frac{\sum_{i=1}^{15}A_{i}}{225A_{T}}$$

where $A_{i}$ is the total pore area in the *i* th microscopic image, $A_{j}$ the area of one certain pore, $D_{j}$ the equivalent diameter of the corresponding pore, $A_{T}$ the area of each microscopic image, and A% the total pore area ratio of the specimen observed. For each analysis, the test surface was analyzed three times starting in a different corner of the sample. By testing 18 analyses of 6 specimens, the average value of those measurements was adopted.

The morphology of hydration product in cement paste sample was scanned by the JSM-IT500LV instrument (JEOL Ltd.,Shenzhen, China) under an accelerating voltage ranging from 10 kV to 20 kV. The selected samples had a flat and polished surface.

## 3. Results and Discussion

### 3.1. Strength Development

**Figure 3:**
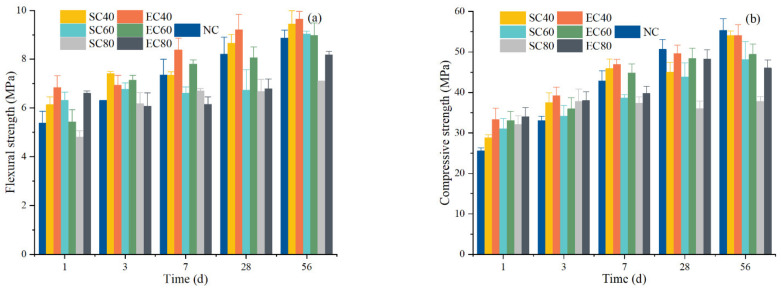
The strength development of the specimens under different curing conditions: (**a**) flexural strength; (**b**) compressive strength.

Figure 3 shows the strength development of the specimens in SC and EC. There are several points worth noting. First, except for the SC80, the strength of SC and EC specimens steadily increased with the curing time. Second, the higher curing temperature, the lower the strength growth rate of specimens at 28 and 56 days. Compared with the NC specimens, the strength of the SC and EC specimens at 1 and 3 days was better, but the strength of some specimens became lower at 7, 28, and 56 days. Finally, the compressive strength of the EC specimens was better than that of the SC specimens under the same curing temperature and age.

The variation of flexural strength of the specimen was basically the same as that of compressive strength. The 1-day compressive strength of NC specimens reached 46.22% of the 56-day NC specimens. All 1-day SC and EC specimens improved by 12.55–33.02% compared to NC-1dand reached 52.02–61.48% of the NC-56d compressive strength. These results indicate that SC and EC could greatly improve the early strength of cement mortar. With the increase of age, the mechanical properties of all groups except the SC80 of specimens were improved. Compared to the NC specimens, the compressive strength of EC40 was increased by 30.45% (1 day), 18.46% (3 days), and 9.7% (7 days), minus 1.98% (28 days) and minus 2.17% (56 days). The compressive strength of SC40 was only 12.55% (1 day), 13.31% (3 days) and 7.24% (7 days), minus 11.06% (28 days) and minus 2.24% (56 days). The mechanical strength results from the other curing regimes show that EC specimens could achieve better mechanical properties than SC specimens. This result indicates that the EC method is a good rapid curing method, which is more conducive to the strength development of the sample compared to the steam curing condition.

### 3.2. Solid Phase Analysis

#### 3.2.1. TG

There is a close relationship between the mechanical properties and the hydration process and products of concrete. In this experiment, cement pastes were employed for solid phase analysis to avoid the interference of sand in the mortar. TG and XRD tests of cement pastes cured in the same regime were performed to analyze the hydration characteristics. Pastes of different ages under the 60 °C curing regime were selected to test the hydration degree and hydration products. The reason for this choice is that 60 °C is the recommended curing regime in steam curing methods [35,36].

**Figure 4:**
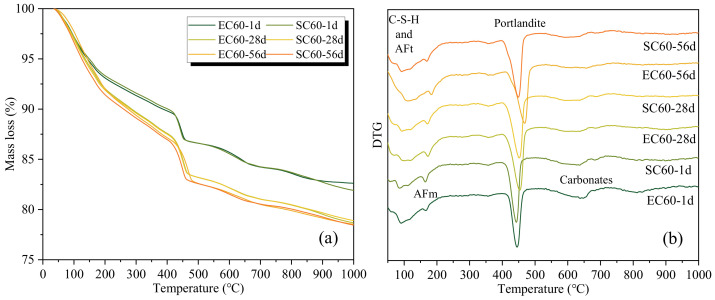
The TG/DTG results of the EC pastes and SC pastes at different ages: (**a**) TG; (**b**) DTG. (AFt means ettringite and AFm means calcium aluminate monosulfate).

For the hydration degree analysis, samples with different curing ages were selected for TG tests, and the results are shown in Figure 4. As can be seen from Figure 4a, there was only a slight difference between the mass loss curves of EC sample and SC sample at the same ages, which indicates that the effect of EC on cement hydration is similar to that of SC. In addition, the mass loss of samples at 28 days was obviously greater than that of 1 day, while it was closer at 56 days. This indicates that the hydration of cement increased significantly within 28 days, but not after 28 days. According to Figure 4b, the mass loss peaks of the EC and SC pastes at different ages were basically the same, which indicates that there was no significant difference in the main hydration products. Moreover, a carbonates peak was found between about 600 °C to 700 °C, which may have been due to the carbonation of the samples by immersion in isopropanol. Based on the results of the TGA, the chemical bound water and portlandite content are listed in Table 3. The difference in chemical bound water and portlandite content between EC and SC were small at the same age, which shows that EC and SC have similar effects on the hydration of cement.

#### 3.2.2. XRD

**Figure 5:**
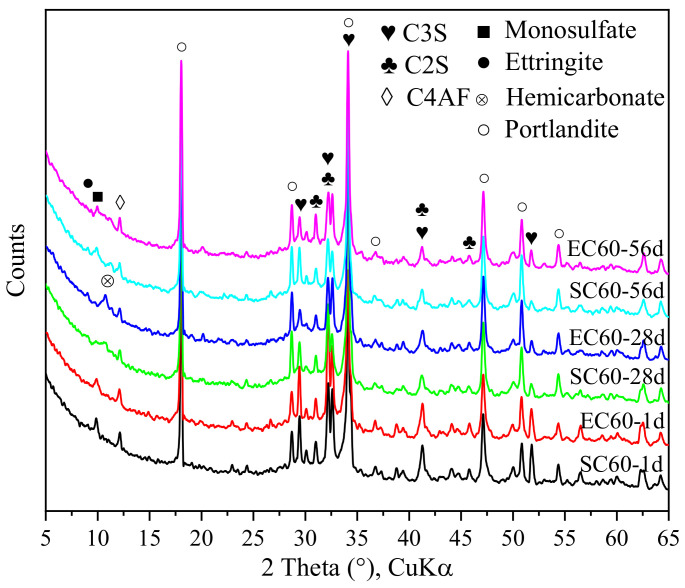
XRD pattern of the EC and SC pastes under different curing ages.

The crystalline phase of the sample was analyzed by XRD, and the results are shown in Figure 5. The diffraction peaks of C_3_S, C_2_S, and C_4_AF can be clearly seen in the XRD pattern, which indicates that these clinker phases did not fully react until 56 days. However, no diffraction peaks about C_3_A were found for 1 day, which shows that C_3_A fully reacted. This may be because the C_3_A reaction was rapid, and the reaction was enhanced by the early high temperature from SC and EC. The main crystalline phase hydration products were portlandite and monosulfate, while ettringite was almost absent. The diffraction peaks of monosulfate decreased in the samples of EC and SC at 28 days, while a diffraction peak of hemicarbonate appeared at about 10.7 (2θ), which may have been due to the carbonization of the samples during storage resulting in generation of hemicarbonate. It is possible that the high temperature curing environment during the early SC and EC processes accelerated the conversion of AFt to AFm. Moreover, a comparison of the EC samples and SC samples reveals that the differences between the XRD patterns of EC and SC samples at different ages were not significant, which further confirms that the effect of EC on cement hydration is similar to that of SC.

### 3.3. Pore Structure

#### 3.3.1. Microscopic Pore Structure

To explain the differences in the mechanical properties of the specimens, the analysis of pore structure was further analyzed.

**Figure 6:**
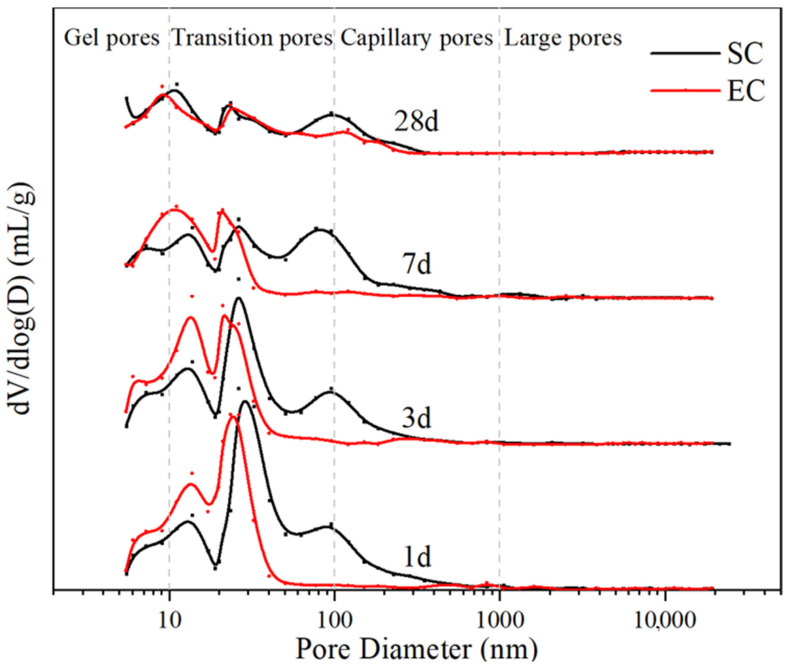
Pore size distribution of the SC60 and EC60 specimens at different ages.

The pore structure of the specimens at different ages under different curing methods was tested by MIP, and the test results are shown in Figure 6. It showed that the number of pores in the range of 20–50 nm gradually decreased for both specimens as the age increased, while the number of pores below 20 nm was gradually increasing. This indicates that with the growth of the age, the specimen was continuously hydrated, refining the original existence of pores. In addition, the EC60 had more pores with the sizes between 10–50 nm than SC60 and fewer pores near 100 nm. Unlike the effect of SC coarsening the pores of cement paste [9,10], EC makes the pore size of cement paste smaller and increases the number of small pores. The data analysis was plotted in Table 4.

**Figure 7:**
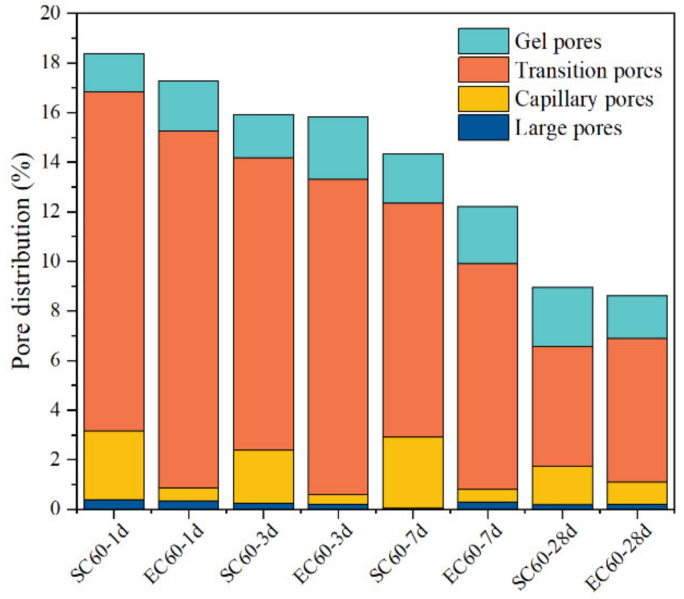
Stacked histogram of the pore size distribution of the specimens under different curing methods.

The porosity results (Table 4) indicate that the difference between EC and SC specimens at the same age was not significant, but the probable pore size results show that the pore size of EC specimens was smaller. This indicates that the pore size inside the EC specimen was smaller than that of the SC specimen for the same porosity. The pores in the cement paste can be divided into four types according to the pore diameters [37,38]: large pores (d ≥ 1000 nm), capillary pores (1000 nm ≤ d < 100 nm), transition pores (10 nm ≤ d < 100 nm), and gel pores (d < 10 nm). Among them, pores greater than 100 nm in diameter are harmful pores, leading to a decrease in mortar strength [39]. The pores distribution data in Table 4 are plotted by pore type in Figure 7. The figure indicates that the number of gel pores of the specimens slightly increased with increasing age. The number of transition pores decreased by filling with dense hydration products. The number of pores above 100 nm was higher for SC60 than for EC60, which indicates that SC caused the coarsening of pores and the formation of more harmful pores, thus affecting the strength development of the specimens.

The MIP results show the difference between the pore structures of the SC and EC specimens. The porosities of the two samples were both decreasing with increasing age, which means that the pores that formed in the early rapid curing regime of the samples continued to hydrate and fill densely under standard curing. In addition, the SC samples had larger porosity than the EC samples, which may be due to the uneven temperature control of the specimens in the curing regime. Uneven high temperature curing led to the formation of temperature gradients inside the sample, and different cement compositions and hydration products produced different expansions, leading to more pore generation. In terms of the most probable pore size, SC was larger than that of EC, indicating that the distribution of pore sizes was affected by different curing methods. Finally, the SC samples formed more harmful pores (>100 nm) than the EC samples, which explains the poorer mechanical properties of SC samples than EC samples. In addition, the gel pores and transition pores of SC samples were less than EC, which means that a more porous pore structure was formed with the same porosity. Therefore, the MIP results indicate that EC is a better curing method than SC, resulting in a better pore structure inside the cement specimens.

#### 3.3.2. Macro/Mesoscopic Pore Image Analysis

**Figure 8:**
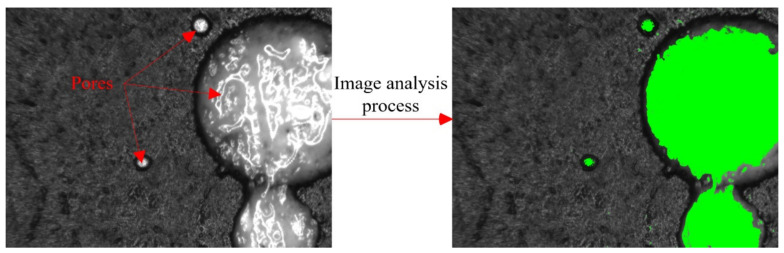
Schematic diagram of the MIA method.

**Figure 9:**
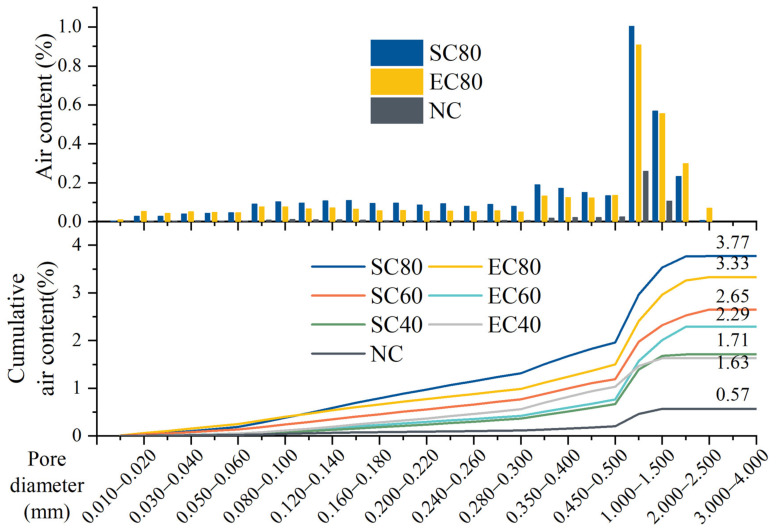
The pore size distribution of the 28-day specimens.

Air voids in hardened concrete, which result from the use of superplasticizer and the inevitably of introducing of air during mixing, are usually larger than 2 μm. The air void structure of the hardened specimens under different curing methods at 28-day can be observed using a microscopic camera. As seen from Figure 8, the air voids on the specimen surface were identified by MIA and marked as green parts by the software. The air distribution and cumulative air content of the 28-day specimens were obtained by calculating the area and size of the green part in all photos by Equations (1)–(3), and the calculation results are shown in Figure 9. For the convenience of observation, the distribution of the number of pores was expressed using only three groups of specimens: SC80, EC80, and NC. The number of air voids in the EC80 specimens with different pore sizes was lower than that in the SC80 specimens. For air void sizes between air void sizes between 0.5 and 2.5 mm, the SC and EC specimens showed a large number of pores compared to NC specimens. The cumulative air content within 0.5 mm of the air voids size of the SC and EC specimens increased by 0.09% and 0.02%, respectively, compared to the NC specimens. However, the cumulative air content within the 2.5 mm air void size of the SC and EC specimens increased by 2.08% and 1.72% compared to 0.57% for the NC specimen. These data demonstrated that the SC and EC specimens had more air voids inside the specimens than NC, which reduced the strength of the specimens. In addition, the SC specimens had more air voids than the EC specimens, which also reduced the strength of the SC specimens. The MIA results matched the mechanical properties results.

The pore evolution of the SC and EC specimens at the microscopic scale and the distribution of air voids at the macroscopic scale were obtained by testing the MIP and MIA. The pore structure of the EC sample was more refined on the microscopic scale than that of the SC, as the formation of gel pores and transition pores was promoted, and the generation of capillary pores was reduced. On the macroscopic scale, the EC specimens produced more air voids than the NC specimens but fewer than the SC specimens. The pore structure data results matched the mechanical property results, which indicated that the EC specimens had better pore structure than the SC specimens.

### 3.4. Discussion

The above results demonstrated that SC and EC accelerated the dissolution of cement minerals in the specimens at the early stage, promoted the formation of portlandites, accelerated the hydration of cement, and enhanced the early mechanical properties of the specimens. The study of pore structure indicates that the pore structure of SC and EC was the main reason for the influence of mechanical properties.

The heat flow transfer characteristic and effect of SC and EC were analyzed to understand the formation of pore structure and temperature evolution of specimens in different curing regimes. Finally, the energy consumption of the two curing methods was discussed.

#### 3.4.1. Temperature Evolution during Curing Regimes

**Figure 10:**
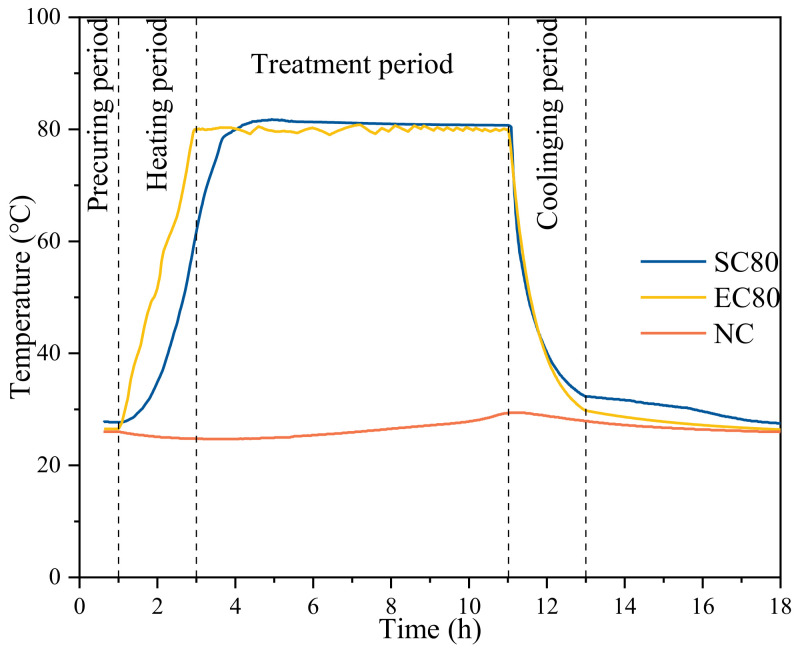
Evolution of temperatures during different curing regimes.

The temperature evolution under different curing regimes is shown in Figure 10. The internal temperature of the SC specimens was lower than the EC specimens during the heating period and higher than the EC specimens in the early part of the constant temperature period.

The SC specimens were heated by the thermal radiation of the water vapor temperature in the water bath tank. The water vapor temperature must increase to raise the temperature of specimen. During the heating period, the SC specimen generated a temperature gradient inside the specimen due to the increasing water vapor temperature and the inefficiency of thermal radiation, which was the main reason for the thermal damage of the specimens. The temperature of EC specimen was controlled by adjusting the voltage during the heating stage. When the EC specimen temperature was lower or higher than the curing regime temperature, the voltage was increased or decreased to the Joule heat inside the specimen.

In the constant temperature period, the SC specimen was affected by the water vapor temperature and its own exothermic hydration, which caused the specimen’s temperature to exceed the control temperature. EC controlled the temperature of specimens by Joule heat, and no overheating occurred. It is necessary to mention that the temperature of the EC specimen fluctuated during the constant temperature period, which was caused by the manual voltage adjustment and could be avoided by the subsequent development of the equipment.

In the cooling period, the specimens cooled down quickly and reached room temperature by opening the box lid for heat dissipation. Since the water bath tank and the insulation box had certain thermal insulation capacity, the temperature of the two specimens was higher than that of the standard NC specimen after the cooling period.

The results indicate that EC was better than SC for the control of specimen temperature. The mechanical properties of the SC and EC specimens were different at the early age. These differences may be attributed to the uncontrolled temperature of the specimens during the constant temperature period. SC is a curing method that uses thermal radiation to heat the specimens. In other words, it suffers the limitations of being a surface-heating method, and it causes a temperature gradient inside the specimens. EC is a volume-heating method that uses the Joule heat produced in the specimens to heat the specimens. In the first 3 h, the environment temperature had reached 40/60/80 °C, but with the hydration exothermic action, the inner temperature of the SC specimen was rising and became uncontrolled. Although the EC specimen experienced this situation, it was easier to control the temperature by adjust the applied voltage. Therefore, the possible reason to explain the different results of mechanical properties in early age was that the temperature of the SC specimens in the curing regime was higher than that of the EC specimens. For the 28-day and 56-day specimens, the mechanical properties of EC60 were higher than those of SC60. This occurred because the EC reduced the microstructural damage during the curing regime, and the unhydrated cement particles in the specimens were better hydrated than those in the SC specimens in the standard curing.

#### 3.4.2. The Heat Flow Transfer Characteristics of Two Curing Methods

**Figure 11:**
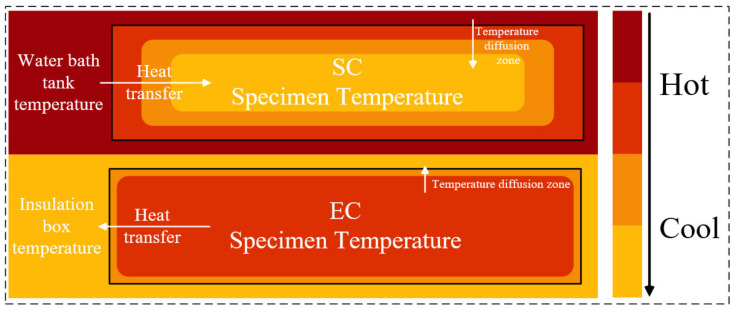
Temperature distribution of the specimens under two curing methods.

The heating principle and heat flow transfer of the two curing methods was analyzed and shown in Figure 11. The SC specimen was heated by heat radiation from water vapor temperature in the water bath tank. The thermal energy first transmitted to the specimen surface and then to its interior, thus forming a multilevel temperature gradient in the SC specimens. The EC specimen was heated up by Joule heat through the flow of AC in the interior of the specimen, using the cement paste with low resistance in the early hydration process. The difference in heat transmission between SC and EC leads to a different heat flow transfer characteristic. For example, the temperature control capability of SC and EC is different. The temperature control capability of SC is mainly determined by the heat transfer efficiency. Cement and concrete are poor conductors of heat transfer and have defects for thermal transfer. The temperature control ability of EC is mainly determined by the resistivity of the cement-based material. The low resistivity of fresh pastes and the adjustable voltage constitute an excellent control capability. Meanwhile, SC is a surface-heating method, and EC is a volume-heating method. The direction of heat transfer determines the temperature gradient inside the specimen. From the surface to the interior of the SC specimens, the temperature transfer forms a multilayer temperature gradient, resulting in defects caused by different temperature expansion of the material. EC specimens generate heat internally, and the specimens undergo an overall heating by Joule heat, reducing the effect of temperature gradient. In addition, the curing efficiency of SC is poorer than that of EC. From the perspective of energy conversion, SC is an indirect curing method by converting water vapor temperature to specimen temperature, while EC is a direct and fast curing method that heats the whole specimen through Joule heat.

After explaining the heat flow transfer characteristic of the two curing methods, SEM was used to observe the specimens cured by the two curing methods. Thus, the morphology of the hydration products was analyzed.

**Figure 12:**
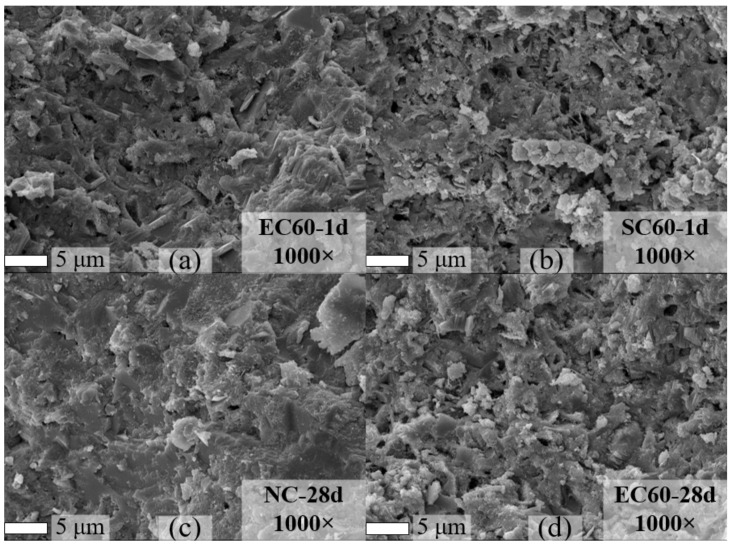
SEM images of the specimens: (**a**) EC sample at 1 day; (**b**) SC sample at 1 day; (**c**) standard curing sample at 28 days; (**d**) EC sample at 28 days.

Figure 12 demonstrates the overall morphology of the microstructure by magnifying the hydration product at 1000×. It can be found that the EC sample of 1-day promoted the dense hydration product with some pores, while the SC sample had more loose hydration products on this basis. In the test of 28-day sample, the morphology of hydration phases of NC and EC samples was shown. It can be found that the samples were dense, and the EC sample had more pores.

#### 3.4.3. Energy Consumption

Based on this investigation, EC is a curing method with easy temperature control and good curing quality. For both curing methods, energy consumption is an important criterion for judging the greening of production. In this paper, the energy consumption of SC and EC was calculated for each group of data collected from 24.4 cm × 4 cm × 16 cm cement mortar specimens. For the EC specimens, the current and voltage values of each specimen recorded by the paperless recorder were used to calculate the total energy consumption. The current values of the water bath tank were collected by the paperless recorder for calculation for SC specimens.

The energy consumption and power of SC and EC were calculated using Equations (5) and (6). To facilitate the comparison of the energy consumption difference between the two test methods, this calculation was only used to test the energy consumption of the test equipment. The energy consumption was not only limited to the hydration exothermic value of the specimen but also included the heat dissipation of the curing chamber to the surrounding environment. Therefore, the data were only used as semiquantitative analysis. The energy consumed per unit area was calculated by dividing the total energy consumption by the total surface area of the specimens. The surface area of EC specimens was $24\times 40\;\mathrm{mm}\times 150\;\mathrm{mm}=0.144\;m^{2}$ due to the layout of the electrode sheet, and the surface area of SC was $24\times 40\;\mathrm{mm}\times 160\;\mathrm{mm}=0.1536\;m^{2}$. The energy consumption per kilogram was calculated by dividing the total energy consumption by the total weight of the specimens. The weight of EC specimens was that,

(5) W=P×t

(6) P=U×I

where *W* was the energy consumption, *P* was the power, t was the time, *U* was the voltage (the voltage of SC specimen was 220 V, EC specimens were collected by paperless recorder), and *I* was the current recorded by the recorder. The energy consumed per unit area (kWh/m^2^) was calculated by dividing the total energy consumption by the total surface area of the specimens. Energy density (kWh/kg) was calculated by diving the total energy consumption by the total weight of the specimens.

**Figure 13:**
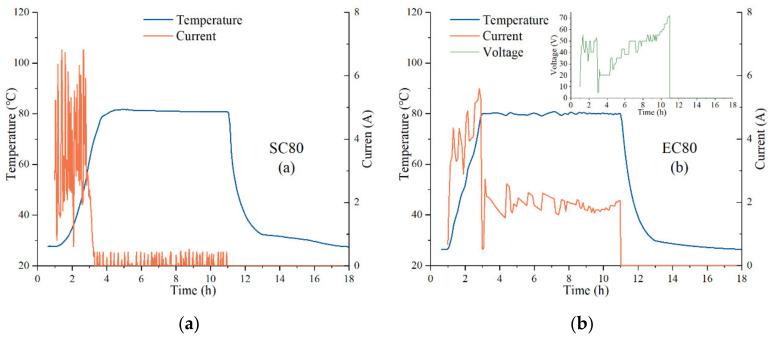
Current and voltage data collected during the 80 °C curing regime: (**a**) SC80 specimens; (**b**) EC80 specimens.

To facilitate the observation of the energy consumption of the curing regime, the most energy-consuming 80 °C curing regime was plotted in Figure 13, which shows the current and voltage values collected by the paperless recorder in the 80 °C curing regime. Whereas Figure 13a displays the operated current of the water bath tank, Figure 13b displays the average current values and real-time voltage values in the EC80 specimens. The results of all curing regimes calculated by Equations (5) and (6) are displayed in Table 5.

SC consumed more energy than EC under the same curing regime, with SC consuming 2.28–9.44-times more energy than EC. The energy consumption of SC was significantly higher than that of EC under the action of the equipment used in this paper, indicating that EC is a low-energy curing method. The energy consumption per unit area of SC was 8.62-, 2.91-, and 2.13-times higher than that of EC at the 40 °C, 60 °C, and 80 °C curing regime, respectively. These results are different from the literature [17], as this test is considered as a long-time temperature control curing, and the electrical or thermal energy was used to control the temperature of the specimen. In addition, the energy consumption mentioned in the literature [17] only applied the same voltage to the specimen. The resistivity of the specimen rose sharply due to the continuation of hydration, which led to a sharp decrease in electrical power reducing energy consumption. To control the temperature of the specimen, this paper promoted the Joule heat generation of the specimen with rising resistivity by increasing the voltage, which greatly consumed the electric power. This was the reason why the energy consumption per unit area in this paper is higher than that in the literature [17], and also shows that SC and EC are more energy-consuming under the long-time temperature-controlled curing regime.

**Figure 14:**
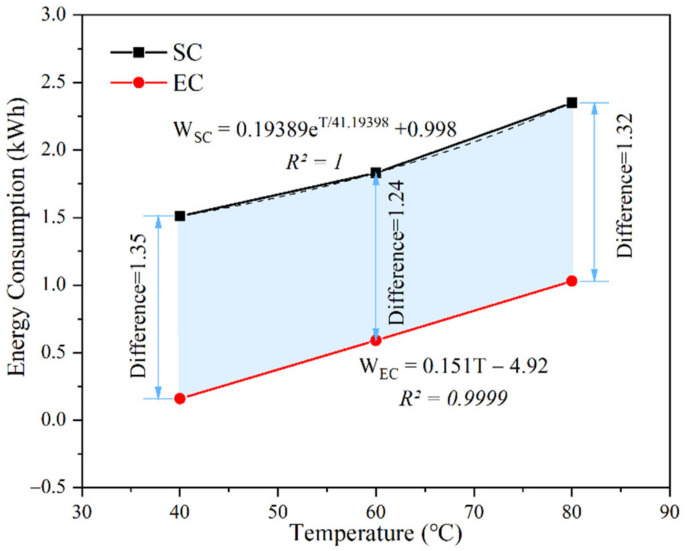
Correlation between the energy consumption and temperature in SC and EC.

The energy density of EC was similar to the results calculated in the literature [22], but the data changed due to the different time of the constant temperature period. This indicates that EC is a curing method with less energy consumption than SC, especially in the 40 °C curing regime where the difference in energy consumption between the two methods could be clearly seen. The energy consumption and temperature correlation were plotted in Figure 14, demonstrating the total energy consumption for SC and EC at different curing regimes, as well as the trend lines and correlation coefficients *R^2^*.

The energy consumption of SC was always higher than that of EC, which was caused by the heat flow transfer characteristic of SC. The energy was first used to heat the water in the water bath tank and then to heat the specimen through the water vapor temperature, which was indirect heating. The heat flow transfer characteristic of EC was to raise the temperature of the specimen through Joule heat, which was a direct heating that could raise the temperature of the specimen quickly and effectively. In addition, the energy consumption of SC needed to be analyzed with an exponential function, which means that, as the temperature of the curing regime increases, the energy consumed would increase exponentially, unlike the linear relationship between the energy consumption of EC, which was proportional to the temperature of the specimen.

The results of the energy consumption analysis show that EC is a lower energy consumption curing method than SC, and the directness and greenness of the volume heating proved that EC is an excellent curing method to harden the cement-based materials quickly and improve the early mechanical properties.

## 4. Conclusions

In this paper, the effects of SC and EC on the mechanical properties and microstructure of cement-based materials were investigated through comparative experiments. The differences in mechanical properties were explained by the hydration degree and products of the cement pastes as well as the pore structure. The energy consumption of the SC and EC regime was also analyzed. The final conclusions were obtained as follows:

1. The compressive strength of both the SC and EC samples increased with the increase of the curing temperature at 1 day, but there was an opposite trend at 28 and 56 days. In addition, the early and late strength of the EC samples were both higher than that of the SC samples. These results indicate that both EC and SC were favorable to the development of early strength but not favorable to the continued growth of later strength, and EC was more favorable to improve the early strength of the samples and alleviate the decrease of later strength.
2. Based on the solid phase analysis, it was found that the degree of hydration and hydration products of EC was basically the same as that of SC, which presented that the influence of EC on the hydration of cement was similar to that of SC. Moreover, the capillary pore structure and air voids distribution of the EC samples were finer than those of the SC samples. These results suggest that the possible reason for the better strength development of the EC samples is the improved microstructure of the hydration products rather than hydration.
3. With the increase of the curing temperature from 40 °C to 80 °C, the energy consumption of SC increased from 2.13 times of EC to 8.62 times, which demonstrates that the energy consumption of SC was much higher than that of EC. Overall, EC can achieve better accelerated curing effects of the mortar than SC with lower energy consumption.

## Figures and Tables

**Table 1:** Oxides composition of the cement and mineral composition of the clinker (wt%).

SiO_2_	Al_2_O_3_	Fe_x_O_y_	CaO	MgO	SO_3_	LOI	C_3_S	C_2_S	C_3_A	C_4_AF
20.58	5.03	3.38	63.32	2.01	2.06	1.76	57.55	17.82	7.54	11.19

Notes: LOI represents loss of ignition; C_3_S, C_2_S, C_3_A, and C_4_AF represent tricalcium silicate, dicalcium silicate, tricalcium aluminate, and tetracalcium aluminoferrite, respectively.

**Table 2:** The parameters of the different curing methods for the specimens.

Group	Curing Method	Curing Regime (°C)	Cement Pasts
SC40	Steam	40	-
SC60	Steam	60	Casted and cured
SC80	Steam	80	-
EC40	Electric	40	-
EC60	Electric	60	Casted and cured
EC80	Electric	80	-
NC	Normal	20	Casted and cured

**Table 3:** The chemical-bound water and portlandite of samples obtained by the TG test (%).

	SC60-1d	EC60-1d	SC60-28d	EC60-28d	SC60-56d	EC60-56d
Chemical bound water	13.77	13.71	17.21	17.23	17.85	17.82
Portlandite content	15.26	14.35	19.24	19.65	19.68	20.43

**Table 4:** The pore characteristics of the specimens under different curing regimes.

Specimen No.		SC60 -1d	EC60 -1d	SC60 -3d	EC60 -3d	SC60 -7d	EC60 -7d	SC60 -28d	EC60 -28d
Porosity/%		18.37	17.26	15.92	15.83	14.34	12.21	8.96	8.61
Probable pore size/nm		26.30	23.41	26.30	21.10	13.74	11.06	11.06	9.06
Pores distribution/%	<10nm	1.53	2.02	1.75	2.53	1.99	3.78	2.38	1.70
	10–100nm	13.67	14.38	11.79	12.71	9.45	8.23	4.84	5.81
	100–1000nm	2.77	0.52	2.11	0.39	2.46	0.50	1.54	0.88
	>1000nm	0.39	0.35	0.27	0.21	0.06	0.30	0.20	0.22

**Table 5:** Energy consumption of steam curing versus direct electric curing.

Curing Method	Energy Consumption (kWh)	Energy Consumed per Unit Area (kWh/m^2^)	Energy Density (kWh/kg)
SC80	2.35	15.28	0.162
EC80	1.03	7.18	0.076
SC60	1.83	11.93	0.126
EC60	0.59	4.10	0.043
SC40	1.51	9.83	0.104
EC40	0.16	1.14	0.012

## Data Availability

Data is contained within the article.
